# Role of Redox Signaling in Neuroinflammation and Neurodegenerative Diseases

**DOI:** 10.1155/2013/484613

**Published:** 2013-12-24

**Authors:** Hsi-Lung Hsieh, Chuen-Mao Yang

**Affiliations:** ^1^Department of Nursing, Division of Basic Medical Sciences, Chang Gung University of Science and Technology, Taoyuan, Taiwan; ^2^Department of Physiology and Pharmacology and Health Aging Research Center, College of Medicine, Chang Gung University, 259 Wen-Hwa 1st Road, Kwei-San, Taoyuan, Taiwan

## Abstract

Reactive oxygen species (ROS), a redox signal, are produced by various enzymatic reactions and chemical processes, which are essential for many physiological functions and act as second messengers. However, accumulating evidence has implicated the pathogenesis of several human diseases including neurodegenerative disorders related to increased oxidative stress. Under pathological conditions, increasing ROS production can regulate the expression of diverse inflammatory mediators during brain injury. Elevated levels of several proinflammatory factors including cytokines, peptides, pathogenic structures, and peroxidants in the central nervous system (CNS) have been detected in patients with neurodegenerative diseases such as Alzheimer's disease (AD). These proinflammatory factors act as potent stimuli in brain inflammation through upregulation of diverse inflammatory genes, including matrix metalloproteinases (MMPs), cytosolic phospholipase A_2_ (cPLA_2_), cyclooxygenase-2 (COX-2), and adhesion molecules. To date, the intracellular signaling mechanisms underlying the expression of target proteins regulated by these factors are elusive. In this review, we discuss the mechanisms underlying the intracellular signaling pathways, especially ROS, involved in the expression of several inflammatory proteins induced by proinflammatory factors in brain resident cells. Understanding redox signaling transduction mechanisms involved in the expression of target proteins and genes may provide useful therapeutic strategies for brain injury, inflammation, and neurodegenerative diseases.

## 1. Introduction

In general, inflammation is a protective response to various cell and tissue injuries. The purpose of this process is to destroy and remove the detrimental agents and injured tissues, thereby benefiting tissue repair. When this helpful response is uncontrolled, the effect initiates excessive cell and tissue damages that result in destruction of normal tissue and chronic inflammation [1–3]. Moreover, the brain inflammatory diseases, including Alzheimer's disease (AD) and Parkinson's disease (PD), are characterized by “redox state” imbalance and chronic inflammation, a major cause of cell damage and death. Reactive oxygen species (ROS) are widely recognized as key mediators of cell survival, proliferation, differentiation, and apoptosis [4, 5]. Excessive production of ROS (termed “oxidative stress”) by mitochondria and NADPH oxidase (Nox) is usually thought to be responsible for tissue injury associated with a range of brain injury, inflammation, and degenerative diseases such as AD [5–8]. Moreover, many of the well-known inflammatory target proteins, including matrix metalloproteinase-9 (MMP-9), cytosolic phospholipase A_2_ (cPLA_2_), cyclooxygenase-2 (COX-2), inducible nitric oxide synthase (iNOS), and adhesion molecules, are associated with oxidative stress (ROS generation) induced by proinflammatory factors such as cytokines, peptides, infections, and peroxidants [3, 5, 9]. Brain cells, especially neuroglial cells, are susceptible to the injurious effects of oxidative stress. Several studies have shown that brain cells like microglia and astrocytes induce and release diverse inflammatory mediators in response to oxidative stress [9–11]. In addition, ROS act as a critical signaling molecule to trigger inflammatory responses in central nervous systems (CNS) through the activation of the redox-sensitive transcription factors, including nuclear factor-*κ*B (NF-*κ*B) and activator protein-1 (AP-1) [5, 9]. Thus, this review will focus on many general aspects of oxidative stress regulation and summarize the current progresses regarding the occurrence and effects of redox signals on CNS and their involvement in the expression of inflammatory target proteins in response to proinflammatory factors during brain inflammation. Moreover, the pharmacological interventions which protect against oxidative stress-induced neuroinflammation and neurodegenerative diseases will be discussed.

## 2. Role of Neuroglial Cells in CNS Physiological and Pathological Events

CNS consists of neurons and glial cells. Among glial cells, astrocytes constitute nearly 40% of the total CNS cell population in the adult human brain, and they maintain homeostasis in normal CNS. Astrocytes have also been proposed to exert a wide range of functions including guidance of the development and migration of neurons during brain development, production of growth factors, maintenance of the integrity of the blood-brain barrier (BBB), and participating in the immune and repairing responses to disease and brain injury [12, 13]. Microglial cells represent resident brain macrophages and can be transformed into activated immunocompetent antigen-presenting cells during the pathological process. An increased number of activated microglial cells have consistently been reported in PD, which may have a deleterious effect on dopaminergic neurons [14]. Astrocytes, as well as microglia, display an array of receptors involved in innate immunity, including Toll-like receptors (TLRs), nucleotide-binding oligomerization domains, double-stranded RNA dependent protein kinase, mannose receptor, and components of the complement system [10]. One common feature of a variety of neurodegenerative disorders is the presence of a large number of activated glial cells including astrocytes and microglia that involve the changes of morphology and expression of many inflammation-related proteins. Gliosis, especially astrogliosis, is characterized by astrocytic proliferation, extensive hypertrophy of the cell body, and functional changes, when stimulated with various factors including lipopolysaccharide (LPS), interleukin-1*β* (IL-1*β*), and tumor necrosis factor-(TNF-*α*) [15, 16].

**Figure 1:**
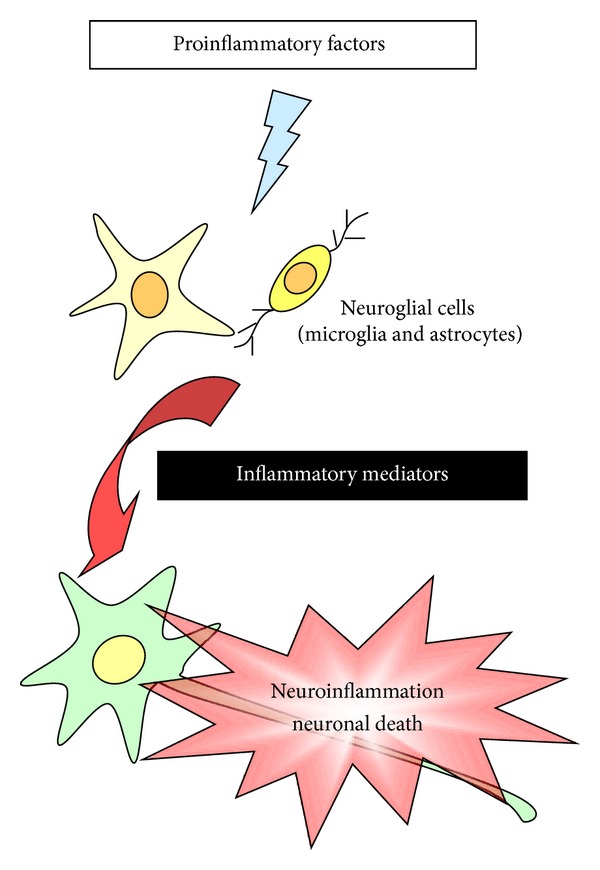
Schematic presentation of the interaction of the brain cells, including neurons and glial cells. In the central nervous system (CNS), proinflammatory factors induce the expression of various inflammatory mediators in neuroglial cells, particularly microglia and astrocytes. These induced inflammatory mediators from glial cells may cause the neuroinflammation or neuronal death, and then leading to neurodegenerative disorders.

Moreover, the cell-cell interactions between glial cells and neurons may be important in the regulation of brain inflammation and neurodegeneration. Many recent reports implicate that inflammation contributes to a wide variety of brain pathologies, apparently killing neurons via glia [10, 11, 17]. Thus, the activated glial cells, especially microglia and astrocytes, are thought to play a critical role in the pathogenesis and progression of neurodegeneration (Figure 1). Previously, many reports have shown that microglial cells may be a major inflammatory cell of the brain [14]. The activated microglia produce several inflammatory mediators including COX-2/prostaglandins (PGs), iNOS/nitric oxide (NO), or cytokines as well as neurotoxic substances, which are thought to be responsible for brain injuries and diseases including trauma, AD, and neural death due to the exposure of LPS, interferon-*γ*, or *β*-amyloid [18, 19]. Although most studies have demonstrated that microglial cells play an important role in neuroinflammation and neurodegeneration, accumulating evidence has also demonstrated the characteristic changes of astrocytes in neurodegenerative diseases such as dementia [10, 11, 20]. Recently, we have demonstrated the upregulation of several inflammatory mediators including MMP-9, cPLA_2_, COX-2, iNOS, and oxidative stress by various proinflammatory factors such as cytokines (e.g., IL-1*β*), peptides (e.g., bradykinin (BK) or endothelin-1 (ET-1)), infections (e.g., bacteria or virus), and peroxidants (e.g., oxidized low-density lipoprotein (oxLDL)) in rat brain astrocytes [21–29]. More recent data indicated that multiple factors including ROS, MMP-9, and heme oxygenase-1 (HO-1)/carbon monoxide (CO) from BK-challenged brain astrocytes may contribute to the neuronal cell apoptosis [30]. Together these results implicate that activated neuroglial cells, especially astrocytes, play a key role in the pathogenesis of the CNS inflammation leading to neurodegenerative diseases (Figure 1).

## 3. Role of Oxidative Stress (Redox Signaling) in the Brain Inflammation and Neurodegenerative Diseases

**Figure 2:**
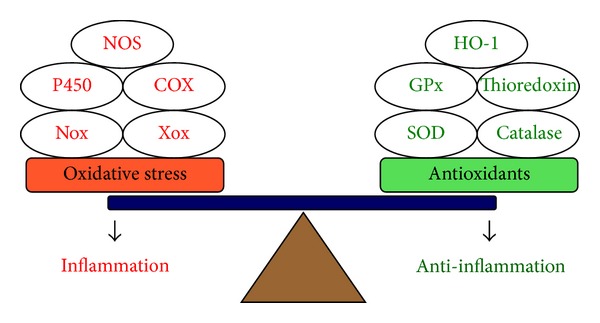
Oxidative stress and antioxidants imbalance in inflammation. In inflammation, the balance appears to be tipped in favor of increased oxidative stress by various specialized enzymes, including Nox, Xox, P450, COX, or NOS, either because of excessive ROS release or inflammatory mediators leading to the amplification of the proinflammatory effects. In contrast, induction of several antioxidants, such as SOD, catalase, GPx, thioredoxin, or HO-1, may reduce ROS generation and attenuate the inflammatory response (anti-inflammation). Nox: NADPH oxidase; Xox: Xanthine oxidase; P450: P450 enzyme; COX: cyclooxygenase; NOS: nitric oxide synthase; SOD: superoxide dismutase; GPx: glutathione peroxidase; HO-1: heme oxygenase-1.

In CNS inflammation, various proinflammatory factors may cause the development of an oxidative stress and antioxidants imbalance, which induces redox signal-dependent expression of genes for inflammatory mediators or protective antioxidants (Figure 2). The oxidative stress (i.e., ROS and reactive nitrogen species (RNS)) is produced by various enzymatic reactions and chemical processes or directly inhaled. ROS that are particularly responsible in oxidative stress include superoxide anion (O_2_^∙−^), hydrogen peroxide (H_2_O_2_), and hydroxyl radical (^∙^OH). Furthermore, the RNS include nitric oxide (NO) and peroxynitrite (ONOO^−^). These oxidative stresses (i.e., ROS/RNS) are essential for many physiological functions at low concentrations [2–6] and killing invading microorganisms [31]. However, several lines of evidence have suggested that the pathogenesis of human diseases is attributed to increased oxidative stress [2, 31]. Moreover, oxidative stress has been shown to mediate the pathogenesis of neurodegenerative diseases, including PD [6], AD [32], and cerebrovascular disorders such as stroke [31]. There are several major sources of ROS/RNS generation in the cells, including Nox, Xanthine oxidase (Xox), P450 enzymes, COX, and NOS (Figure 2), which contribute to several physiological and pathological functions including brain inflammation and neurodegeneration [8]. The physiological role of ROS/RNS (along with O_2_^∙−^ and NO) also extends to the control of vascular tone in the brain, which is tightly modulated by the metabolic activity within neurons [6, 33]. Particularly in the brain, even small redox imbalances can be deleterious. Recently, accumulating evidence attributes the cellular damage in the CNS degenerative disorders to oxidative stress [5–9], suggesting that oxidative stress is an early event in AD [32]. Oxidative stress may be responsible for brain inflammatory disorders, which cause deleterious effects during CNS pathogenesis [34]. Furthermore, several reports have shown that ROS levels are increased with age in several major organs including brain [32]. Abnormally elevated ROS is implicated in age-related long-term potentiation (LTP) impairment [35]. ROS further induce expression and activation of proinflammatory factors or inflammatory mediators during brain injury and inflammation. Under various pathological conditions, excessive amounts of ROS can damage DNA, lipids, proteins, and carbohydrates leading to impairing cellular functions and enhancing inflammatory reactions [34, 36]. In brains of AD patients, cellular and animal models of AD, the elevated levels of these oxidative stress-modified molecules are also detected [32]. Recently, increasing evidence attributes the cellular damage in neurodegenerative disorders such as AD and PD to oxidative stress that leads to generation of ROS associated with brain inflammatory disorders [2, 6]. Thus, these results indicate that oxidative stress (i.e., ROS production) plays an important role in CNS inflammation and neurodegenerative disorders (Figure 4). 

Oxidative stress activates several intracellular signaling cascades that may have a deleterious effect on the cellular homeostasis. The molecular mechanisms associated with ROS production (e.g., mitochondrial dysfunction and Nox activation) and its influences have been investigated in various models of chronic inflammation and neurodegenerative disorders [9]. Recently, there are extensive pieces of literature supporting a role of mitochondrial dysfunction and oxidative damage in the pathogenesis of AD [5, 37], and ROS are associated with neuroinflammatory and neurodegenerative processes [9, 17, 32]. Several proinflammatory factors (e.g., LPS and BK) have been shown to induce the expression and activation of various inflammatory mediators via a ROS-dependent manner in brain cells [25, 36]. In microglial cells, ROS, as a major signaling molecule, mediate microglial activation induced by proinflammatory mediators such as A*β* or LPS [38, 39]. However, the roles of oxidative stress that contribute to these events are not well characterized in brain cells including astrocytes. Our recent reports have demonstrated that ROS signals contribute to the expression of many inflammatory genes (e.g., MMP-9) by several proinflammatory factors, including BK [25], LTA [27], and TGF-*β*1 [40] in brain astrocytes. More recent result indicates that ROS generation from BK-challenged astrocytes contributes to neuronal apoptosis through a caspase-3-dependent manner [30]. Although oxidative stress is implicated as a causative factor in neurodegenerative disorders, the signaling pathways linking ROS production with neuronal cell death are not well characterized [6]. Hence, there are several targets and signals that need to be identified and explored for the development of therapeutic strategies in the future.

## 4. Redox Signaling and Proinflammatory Factors in Brain Inflammation and Neurodegenerative Diseases

The senile and neuritic plaque of AD are accompanied by inflammatory responses in activated glial cells (i.e., astrocytes and microglia). In CNS, several cytokines and inflammatory mediators produced by activated glia have the potential to initiate or exacerbate the progression of neuropathology [41]. Moreover, traumatic injury to CNS results in the production of inflammatory cytokines via intrinsic (brain cells) and extrinsic means (by infiltrating macrophages and other leukocytes). The expression of many inflammatory mediators including cytokines, MMPs, cPLA_2_, COX-2, and iNOS has been shown to be regulated by various extracellular stimuli such as proinflammatory cytokines (e.g., IL-1*β* and TNF-*α*), peptides (e.g., BK, ET-1, and A*β*), infections (e.g., bacteria and virus), peroxidants (e.g., oxLDL and H_2_O_2_), and other stresses (e.g., TGF-*β*) in neuronal and neuroglial cells [4–9, 42] (Figure 4).

### 4.1. Cytokines

IL-1*β* and TNF-*α* are two of the inflammatory cytokines significantly elevated in neurodegenerative diseases such as AD, and they play a central role in initiating and regulating the cytokine cascades during inflammatory responses [43]. IL-1*β* is a pleiotropic cytokine and classified as a dominant injury biomarker. Furthermore, several studies have shown that the level of IL-1*β* is elevated in the cerebrospinal fluid (CSF) of patients with AD, traumatic brain injury [44], and stroke [45]. Thus, IL-1*β* plays an important role in both acute and chronic neurodegenerative diseases. The effects of IL-1*β* on ROS generation have been reported to be associated with brain inflammatory disorders, cancers, and myocardial remodeling [46, 47]. ROS generation by IL-1*β* leads to the expression of several inflammatory genes like MMP-9 which may increase BBB permeability, recruit immune cells infiltrating through BBB into the tissues, and subsequently result in brain inflammation and edema during brain injury [6, 34]. ROS may also act as an inflammatory signaling factor mediated microglial activation induced by IL-1*β* [39]. Moreover, in culture of glia/neuron, IL-1*β* induces neurotoxicity through the release of free radicals [48]. In addition, TNF-*α* is also produced in response to oxidative stress and A*β*. In brain, TNF-*α* is produced by microglia and its overproduction has been linked with neuronal cell death [49]. These studies indicate that cytokines, especially IL-1*β* and TNF-*α*, contribute to the CNS inflammation and neurodegenerative diseases through redox signalings.

### 4.2. Peptides

AD is defined by progressive impairments in memory and cognition and by the presence of extracellular neuritic plaques (A*β*) and intracellular neurofibrillary tangles (tau protein) [5, 32]. Among these molecules, A*β* is an insoluble fibrous protein and aggregates sharing specific structural traits. It arises from at least 18 inappropriately folded versions of proteins and polypeptides present naturally in the body. The misfolded structures alter their proper configuration such that they erroneously interact with other cell components forming insoluble fibrils. A*β* has been associated with the pathology of more than 20 human diseases including AD. Abnormal accumulation of amyloid fibrils in brain may play a role in neurodegenerative disorders. Although A*β* peptide is neurotoxic species implicated in the pathogenesis of AD, mechanisms through which intracellular A*β* impairs cellular properties and produces neuronal dysfunction remain unclear. Accumulating evidence has indicated that A*β* can stimulate the production of free radicals [50]. Interestingly, intracellular A*β* is present in mitochondria from brains of transgenic mice with targeted neuronal overexpression of mutant human amyloid precursor protein and AD patients. Importantly, mitochondria-associated A*β*, principally A*β*_1–42_, was detected as early as 4 months, before extensive extracellular A*β* deposits [51]. Moreover, activation of Nox by A*β*_1–42_ results in ROS production in rat primary culture of microglial cells [52]. In mouse models of plaque formation, oxidative stress occurs prior to A*β* deposition in a Tg2576 APP transgenic mice [53]. Moreover, increased levels of oxidative damage occur in individuals with mild cognitive impairment (MCI), which is often believed to be one of the earliest stages of AD [54]. Additionally, glial HO-1 expression in the MCI temporal cortex and hippocampus is also significantly greater than that of the nondemented group [55]. These results support A*β*-induced redox signaling serving as an early event that leads to the development of the CNS pathological features such as AD. Moreover, glial cells may play a key role in the events.

In addition to A*β* peptide, BK and related peptides are produced and released during trauma, stroke, and neurogenic inflammation [56]. All these pathological processes may be involved in tissue remodeling, which were regulated by MMPs. Moreover, astrocytes possess receptors for numerous transmitters such as glutamate and BK [57]. These peptides mediate several inflammatory responses including increasing vasodilatation and vascular permeability, promotion of fluid secretion and ion transport, and eliciting itching and pain at the sites exposed to noxious stimuli. Thus, the elevated level of BK plays a key role in the initiation of inflammatory responses in target tissues, including CNS. It is well established that BK interacts with two BK receptor subtypes, including BK B1 and B2 [58]. Astrocytes are known to express B2-type BK receptors and this type of receptors is found only on astrocytes type 1 [57]. The B2 BK receptor is a heterotrimeric G-protein-coupled receptor (GPCR) that can be coupled to intracellular signaling molecules via interaction with G_q_ protein [59]. Activation of BK receptors stimulates intracellular signaling molecules, including Ca^2+^, PKCs, and MAPKs, in several cell types including astrocytes [57–59]. Activation of these signaling pathways may lead to cell survival, proliferation, differentiation, and the expression of several inflammatory genes such as iNOS and MMP-9 [36, 60]. During brain injury, BK has been shown to induce the expression of several inflammatory genes by increasing ROS production [6, 34]. Moreover, Nox is expressed in astrocytes and contributes to ROS generation [61, 62]. In brain astrocytes, BK induces the expression of several inflammatory genes like MMP-9 by ROS-dependent signaling pathways [25]. Moreover, ROS released from BK-challenged brain astrocytes cause neuronal cell apoptosis [30]. These pieces of literature suggest that BK plays an important role in brain inflammation and neurodegenerative disorders.

Endothelial cells are known to produce vasotone mediators such as endothelins (ETs) and NO to maintain hemodynamic responses. The ETs are 21-amino acid vasoconstricting peptides produced primarily in the endothelium, which play a key role in vascular homeostasis and have been implicated in brain inflammatory diseases. Among the ET family, the bioactivity of ET-1 is mediated through potent vasoconstrictor and proinflammatory action in vascular diseases, including the heart, circulation system, and brain [63–66]. Two types of ET receptors, ET type A (ET_A_) and type B (ET_B_), are responsible for ET-1-triggered biological effects, which are mediated via G-protein-dependent processes [63–65]. In CNS, ET-1 also plays a substantial role in the normal development and CNS diseases. Both endothelial cells and astrocytes are potential sources of ET-1 release in response to hypoxic/ischemic injury of the brain [66]. On astrocytes, the ET_B_ receptors are predominantly expressed and modulate postinjury responses of astrocytes in CNS [67]. Circumstantial evidence has further demonstrated that overexpression of ET-1 has deleterious effects on astrocytes in ischemic brain [68]. Similarly, ET-1 causes hypertrophy of ET_B_/GFAP-immunoreactive astrocytes, a typical characteristic of astrogliosis, in the normal optic nerve, leading to glial scar formation following CNS injury [68]. Endothelial ET-1 induces cytokine production such as IL-1*β* released by astrocytes, which directly contributes to BBB breakdown during CNS inflammation [69]. These findings further imply the involvement of ET-1 in the CNS inflammation and diseases.

### 4.3. Infections

Bacterial infections have been shown to be involved in brain inflammation [70]. A well-known endotoxin from Gram-negative bacteria, LPS, regulates the expression of inflammatory proteins associated with inflammatory diseases. Many studies have also shown that ROS are the major signaling molecule which mediates microglial activation induced by inflammatory mediators, including LPS [71]. However, the signaling mechanisms of which activated brain cells in response to Gram-positive bacterial infection remain undefined. Gram-positive bacterial infections of CNS occur in bacterial meningitis and brain abscess, being localized to the membranes surrounding the brain and in its parenchyma [72]. Lipoteichoic acid (LTA), an amphiphilic polymer, is embedded in-cell wall of Gram-positive bacteria [73]. The Gram-positive bacterium *Streptococcus pneumoniae* is the most common cause of acute bacterial meningitis worldwide [74], revealing a close relationship between LTA challenges and CNS diseases. For the initiation of LTA signaling, TLRs are believed to be responsible for LTA recognition challenged by Gram-positive bacteria such as *Staphylococcus aureus* and *Streptococcus pneumoniae* [75]. Upon binding to TLR heterodimers (i.e., TLR2/TLR1 or TLR2/TLR6 complex), LTA exerts a sequential activation of members of IL-1 receptor-associated kinase (IRAK) family and tumor necrosis factor receptor-associated factor 6 (TRAF6), mediated by a TLR adaptor protein MyD88. Ultimately, TLR signalings activate MAPK family and NF-*κ*B, leading to modulation of gene expression of cytokines and other inflammatory proteins [76]. Among the diverse cell types in CNS, glial cells such as astrocytes and microglia are regarded as targets in Gram-positive bacterial infection [77–79]. Several lines of evidence suggest a causal relationship between LTA challenges and the CNS diseases, which involves glial activation and TLR2 signalings [77–79]. TLR signalings in astrocytes have been shown to be involved in inflammatory responses in CNS [80], accompanied with upregulation of genes with inflammatory and proapoptotic effects [81]. The pathogenic progression involves glial activation and TLR2 signalings stimulated by LTA, which are linked to inflammatory neurodegeneration [82]. Additionally, LTA exhibits detrimental effects on brain cellular functions, including induction of apoptosis, production of oxidative stresses, and disruption of BBB following group B *Streptococcus* or *Staphylococcus aureus* challenge in CNS [82]. Although the effects of LTA on ROS generation have been reported in several cell types such as renal diseases [83], LTA-induced brain cell responses through the ROS signals are not well characterized. Recent report indicates that LTA-induced MMP-9 expression is mediated through Nox2-derived ROS generation in brain astrocytes [27]. These data suggest that targeting LTA and its specific signaling components could yield useful therapeutic targets for CNS inflammatory diseases upon infection with Gram-positive bacteria.

Moreover, increasing evidence has shown that viral infections such as Japanese encephalitis virus (JEV) and Enterovirus 71 (EV71) may contribute to several inflammatory responses in CNS [28]. Neurotropic viruses can cause massive neuronal dysfunction and destruction that lead to neurological diseases. EV71, a single-positive-stranded RNA virus, belongs to the Enterovirus B genus of the Picornaviridae family [84]. EV71 and Coxsackievirus A16 (CVA16) are the major causative agents of hand-foot-and-mouth disease (HFMD) that is usually mild exanthematous infection and self-limiting in the young children. However, EV71, but not CVA16, can progress to severe neurological diseases including fatal encephalitis, aseptic meningitis, and fatal neurogenic pulmonary edema [85]. Children under 5 years old of age group are susceptible to these infections and may develop permanent neurological sequelae or even succumb to such disorders [86]. In 1998, an EV71 outbreak infected more than 130,000 children resulted in 78 fatalities. Since then, EV71 infection has recurred every year in Taiwan and EV71 outbreaks have been periodically reported throughout the world, representing a major public health concern particularly in the Asia-Pacific regions including Taiwan, Malaysia, Singapore, Japan, and China [85, 87]. The emerging evidence suggests that ROS affect the interaction between host and viral pathogens. Recently, EV71 has been shown to induce oxidative stress-dependent viral replication in human neuroblastoma SK-N-SH cell line [88]. Similarly, JEV is a single-stranded, positive-sense RNA virus belonging to the family Flaviviridae. JEV is transmitted between animals and humans by culex mosquitoes [89]. After the bite of an infected mosquito, JEV amplifies peripherally producing transient viremia before entering into CNS [89]. The principal target cells for JEV are localized in CNS, including neurons and astrocytes [90]. Several lines of evidence suggest that JEV frequently causes severe encephalitis in the world, especially in Eastern and Southeastern Asia. The infection with JEV is characterized by clinical manifesting with fever, headache, vomiting, signs of meningeal irritation, and altered consciousness leading to high mortality [89, 90]. The generation of ROS plays an important role in diverse cellular functions including signal transduction, oxygen sensing and host defense during infection by viruses such as JEV [91]. In CNS, JEV infection has been shown to upregulate MMP-9 gene expression through ROS-dependent pathways in brain astrocytes [28]. These findings concerning JEV-induced expression of inflammatory genes in brain astrocytes imply that JEV might play a critical role in the brain inflammation and neurodegenerative diseases.

### 4.4. Peroxidants

Oxidative stress may cause production of several peroxidants such as oxidized lipoprotein. Clinical reports reveal that the patients with AD exhibit an increased oxidation of lipoproteins potentially toxic to neurons in CNS [92]. Among these, the oxidized low-density lipoprotein (oxLDL) is a well-known predominantly risk factor of atherosclerosis, which has been reported to participate in the progression of the CNS diseases. In CNS, oxLDL exhibits detrimental effects on brain cell functions, including induction of apoptosis, disruption of capillary homeostasis, and alteration of inflammatory protein activity in various brain cells [93]. Furthermore, in patients with cerebral infarction, oxLDL is present in brain parenchyma and stimulates astrocytes to secrete interleukin-6 [94] and may serve as an indicator to reflect the level of oxidative stress [95]. In brain astrocytes, oxLDL can induce MMP-9 expression and cell migration, which plays a critical role in the progression of inflammatory diseases and remodeling processes in target tissues, including CNS [29, 96]. These findings suggest that peroxidants like oxLDL might play a key role in the progression of the CNS diseases and also that targeting these peroxidants-stimulated signaling components may provide useful therapeutic strategies for brain inflammation and neurodegenerative diseases.

### 4.5. Others

In addition to these well-known factors, there are many factors that may also contribute to neuroinflammatory responses. Among these, TGF-*β* has been implicated to participate in the responses. TGF-*β* binds to two serine/threonine kinase receptors which consist of TGF-*β*RI and TGF-*β*RII. During ligand binding, TGF-*β*RII phosphorylates TGF-*β*RI and activates Smad-dependent intracellular signaling pathways and thus leads to expression of several genes [97, 98]. In addition to activation of Smad-dependent pathways, TGF-*β* can affect several signal transduction pathways in a Smad-independent manner, such as MAPKs [97, 98]. In human gingival and skin fibroblasts, both p38 MAPK and Smad3 cooperate in regulating TGF-*β*-induced MMP-13 expression, whereas ERK1/2 cooperates with Smad3 in regulating connective tissue growth factor expression [99]. Recently, increasing evidence has attributed the cellular damage in neurodegenerative disorders to oxidative stress leading to generation of ROS that are responsible for brain inflammation and neurodegenerative disorders [6, 34]. TGF-*β* can stimulate ROS production, which participates in the expression of diverse inflammatory genes such as MMPs in the processes of several human inflammatory diseases [100]. In brain astrocytes, TGF-*β*1 has been shown to induce inflammatory protein expression via a ROS-dependent manner [40]. These results suggest that TGF-*β*1 may play a key role in the process of brain inflammation and neurodegenerative diseases.

## 5. Role of Redox Signaling in the Regulation of Inflammatory Mediators

Neuroinflammation is an active defensive process against diverse insults, metabolic and traumatic injuries, infection, and neurodegenerative diseases. Although neuroinflammation serves as a neuroprotective mechanism associated with repair and recovery, it can also cause brain damage [101]. However, if inflammation in the brain is chronic or inappropriately controlled, it may become detrimental to neurons, thus representing one of the various pathological insults induced by various proinflammatory factors and by inflammatory mediators in CNS [101]. Experimental and clinical studies have shown that various inflammatory mediators are present in brain, CSF, and blood in brain injury. In particular, the histological analysis of human brain from individuals with brain disorder such as AD or epilepsy of various etiologies strongly suggests the existence of a chronic inflammatory state in the brain almost invariably associated with neuronal loss or reactive gliosis [102]. In experimental models of rodent brain seizures, a variety of inflammatory mediator mRNAs and protein levels are rapidly increased after the induction of seizures, including MMPs (e.g., MMP-9, especially), multiple forms of PLA_2_ (e.g., cPLA_2_), COX-2, NOS (e.g., iNOS), and adhesion molecules (e.g., ICAM-1 and VCAM-1) [102, 103]. After expression of these inflammatory mediators, several CNS damaging factors will be produced such as cytokines shedding by MMPs, arachidonic acid (AA)/PGE_2_ releasing by cPLA_2_/COX-2 system, and NO generation by NOS [102, 103]. Herein, we reviewed the role and mechanism of these inflammatory mediators in the brain inflammation and neurodegeneration and whether oxidative stress plays a crucial role in these events.

### 5.1. Matrix Metalloproteinases

MMPs are a large family of zinc-dependent endopeptidases, which play an important role in the turnover of extracellular matrix (ECM) and pathophysiological processes [104]. To date, 24 MMPs have been identified in mammals. Among these MMPs, some are membrane-type MMPs which are anchored to the cell surface and others are secreted into the extracellular space. In general, MMPs are released as inactive proform MMPs and activated by proteolytic cleavage of the N-terminal domain. In gelatinase subfamily of MMPs (i.e., MMP-2 and MMP-9), the catalytic domain that contains the Zn^2+^ binding site and repeats of fibronectin motifs allowing the ability to bind their major substrate gelatin. MMP-9 (gelatinase B; 92 kDa) is usually low and its expression can be induced by various proinflammatory factors such as cytokines. The other class of gelatinase, MMP-2 (gelatinase A; 72 kDa), is constitutively expressed in several cell types and usually not inducible. In CNS, MMPs, especially, MMP-9 are implicated in several important physiological events, including morphogenesis, wounding healing, and neurite outgrowth [105]. Moreover, upregulation of MMP-9 may contribute to the pathogenesis of several CNS diseases such as stroke, AD, multiple sclerosis, and malignant glioma [105]. Several proinflammatory factors including cytokines, endotoxins, and oxidative stress have been shown to upregulate MMP-9 in astrocytes *in vitro* [106, 107], implying that MMP-9 activity may be regulated by diverse factors in CNS during neuroinflammation. Moreover, many proinflammatory mediators like cytokines and BK induce the expression of MMP-9 during brain injury by increasing ROS production [25, 62]. Recently, upregulated MMP-9 and ROS generation from brain astrocytes have been reported to contribute to neuronal cell death *in vitro* [30]. These studies suggest that upregulation and activation of MMP-9 by proinflammatory factors are mediated through oxidative stress (ROS production) during brain injury and inflammation (Figure 4). Therefore, the inhibition of MMP-9-mediated inflammatory pathways may provide therapeutic strategies to brain inflammation and neurodegenerative diseases.

### 5.2. Cytosolic Phospholipase A_2_

There are three forms of phospholipase A_2_ (PLA_2_) superfamily including the secretory PLA_2_, type IV PLA_2_, also known as cPLA_2_, and calcium-independent PLA_2_ in mammalian cells [108–110]. The secretary PLA_2_ (sPLA_2_) is expressed in a variety of cell types and it has no preference for AA at *sn*-2 position, requires millimolar amounts of Ca^2+^ for activity and is sensitive to sulfhydryl reducing agents, such as dithiothreitol (DTT), and is resistant to heat or acid conditions [109]. The calcium-independent PLA_2_ (iPLA_2_) does not require Ca^2+^ for catalytic activity. The iPLA_2_ prefers plasmalogen substrates and does not appear to have a preference for the type of fatty acid at the *sn*-2 position. The third class is the novel and high molecular weight (85 kDa) cPLA_2_. The cPLA_2_ catalyzes the hydrolysis of the *sn*-2 position of membrane glycerophospholipids, leading to production of free fatty acids and lysophospholipids. This reaction is of particular importance if the esterified fatty acid is AA, which is converted by downstream metabolic enzymes to various bioactive lipophilic compounds called eicosanoids, including PGs and leukotrienes (LTs) [110]. PLA_2_ could be the initial and rate-limiting enzyme in this conversion. The increase in cPLA_2_ activation and expression following external stimuli, including proinflammatory cytokines, growth factors, and microbial toxin, is often observed in several systems [111]. Among these enzymes, cPLA_2_ is the only one that plays a key role in mediating agonist-induced AA release for eicosanoid production in various cell types [112]. Several studies have indicated that cPLA_2_ is constitutively expressed in the cytosol of most resting brain cells and tissues. In brain, cPLA_2_ has been shown to co-localize with glial fibrillary acidic protein (GFAP), a principal marker for brain astrocytes [113]. Moreover, under brain inflammatory and neurodegenerative conditions such as AD, there is an increase in immunoreactivity to cPLA_2_ in astrocytes from the cortex of patients [114, 115]. A variety of proinflammatory factors including IL-1*β*, TNF-*α*, or BK may exert as modulators of cPLA_2_ activity and/or expression in various cell types including astrocytes [23, 111]. Upregulation and activation of cPLA_2_ leading to PGE_2_ production have been implicated in a number of neurodegenerative diseases [111, 114, 115]. Recently, PGE_2_ production and cPLA_2_ activation have also been shown to regulate the CREB-dependent iNOS expression in microglia [116] or cPLA_2_ expression in amnion fibroblasts [117]. However, a series of highly reactive PGs, free fatty acids, lysophospolipids, eicosanoids, platelet-activating factor, and ROS, all generated by enhanced PLA_2_ activity and AA release, participate in cellular injury, particularly in neurodegeneration [118]. Thus, cPLA_2_ seems to function as a crucial upstream regulator of the production of eicosanoids during brain inflammation and is correlated to the process of neurodegenerative diseases (Figure 4). The inhibition of cPLA_2_-mediated pathways may provide a therapeutic strategy to brain inflammation and neurodegenerative diseases.

### 5.3. Cyclooxygenase-2

COX, known as a prostaglandin-endoperoxide synthase, is a rate-limiting key enzyme in the synthesis of PGs. In this process, PLA_2_ catalyzes the release of AA from membrane phospholipids, while COX catalyzes the conversion of AA into PGs [119]. Significant advances have been made in understanding the role of COX in certain biologic processes, including inflammation, angiogenesis, development, and several homeostasis [119]. COX exists in two isoforms: COX-1, which is expressed constitutively under normal conditions in most tissues, mediates regulating normal physiological responses, and controls renal homeostasis, and the inducible COX-2, is not detectable in most normal tissues or resting cells, but its expression can be induced rapidly by a variety of stimuli including cytokines, bacterial or viral infections, and other mediators to produce PGs during inflammation [120]. In addition, COX-2 gene promoter which contains multiple regulatory elements has been shown to be regulated by different transcription factors, including NF-*κ*B, AP-1, and cyclic AMP-response element binding protein (CREB) in various cell types [121]. Previous studies showed that COX-2 immunoreactivity is a characteristic finding in the synovial macrophage of patients with arthritis as well as in other forms of inflammation. Moreover, several lines of evidence have confirmed COX-2 as a major therapeutic target for the treatment of inflammatory disorders such as arthritis [119, 122]. Recently, the mice with homozygous deletion of the COX-2 gene suppress endotoxin-induced inflammation [123]. In brain, expression of COX-2 leads to increased production of prostanoids which are potent inflammatory mediators, and upregulated COX-2 expression has been reported in neurodegenerative disorders [124]. Moreover, upregulation of COX-2 and PGE_2_ release by viral infection such as EV71 have been reported in brain astrocytes and human neuroblastoma cells via diverse signaling pathways [125, 126]. Upregulation of COX-2/PGE_2_ by ET-1 via MAPK-dependent NF-*κ*B pathway in brain microvascular endothelial cells [127]. A recent report also indicates that the ROS-induced COX-2 expression can be found in ALS [128]. However, the expression of COX-2 appears to be strongly induced and activated during AD, indicating the importance of inflammatory gene pathways as a response to brain injury [118]. Thus, COX-2 may play an important role in the development of brain inflammation and neurodegenerative diseases.

### 5.4. Nitric Oxide Synthase

**Figure 3:**
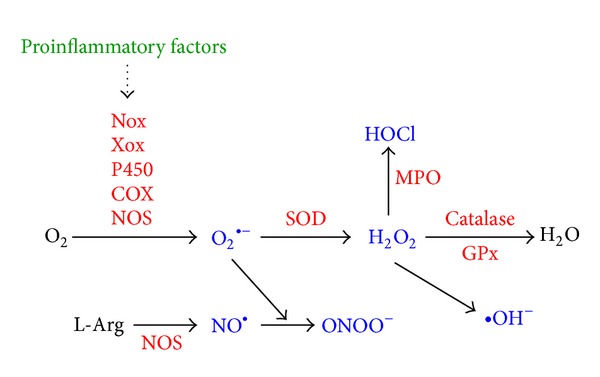
Major pathways of reactive oxygen (nitrogen) species generation and metabolism. Several proinflammatory factors can stimulate O_2_^∙−^ generation through activation of several specialized enzymes, such as the Nox, Xox, P450, COX, or NOS. SOD then converts the O_2_^∙−^ to H_2_O_2_, which is then converted into the highly reactive ^∙^OH or has to be rapidly removed from the system that is generally achieved by catalase or peroxidases, such as the GPx. Further, O_2_^∙−^ can be either converted into ROO^∙^ or can react with NO to yield ONOO^−^. NO is mostly generated by L-Arg via NOS. H_2_O_2_ can be converted to HOCl by the action of MPO. myeloperoxidase. O_2_: molecular oxygen; H_2_O: water; O_2_^∙−^: superoxide radical anion; ^∙^OH: hydroxyl radical; ROO^∙^: peroxyl radical; H_2_O_2_: hydrogen peroxide; ONOO^−^: peroxynitrite; NO: nitric oxide; L-Arg: L-arginine; HOCl: hypochlorous acid.

NO is a free radical that displays diverse bioactivity in various organ systems, including CNS. Depending on the concentration, excess NO levels are implicated in the pathogenesis of CNS diseases including ischemia, trauma, neuroinflammatory, and neurodegenerative diseases [129–131]. Production of NO from L-arginine is catalyzed by NOS. The level of iNOS in healthy brain is undetectable. Accumulating evidence supports the role of iNOS in the pathogenesis of CNS disorders. In CNS, upregulation of iNOS in various cell types, including astrocytes and microglia, is proposed to be the leading source of NO production during neuroinflammation [132]. Furthermore, knockout strategies of iNOS gene protect against focal cerebral ischemia and LPS challenges [133, 134]. iNOS is induced by a variety of stimuli, such as viral and bacterial infections, cytokines, cell-cell contact, and neurotoxins [131]. The consequent product NO reacts with superoxide to form peroxynitrite (ONOO^−^), the most toxic derivative of NO (Figure 3). As for the involvement of NO derivatives in neuropathology, many studies have revealed that the reference of iNOS/NO/ONOO^−^ plays an important role in neurodegenerative disorders [131]. However, following inflammatory insults, reactive astrocytes express iNOS, which causes the neuronal damage associated with cerebral ischemia and/or demyelinating diseases [132]. In CNS, appearance of iNOS in astrocytes is related to several neurodegenerative diseases such as ALS [130] and multiple sclerosis (MS) [129]. These findings imply that astrocytes are the leading regulators in neurodegenerative diseases. Moreover, activation of astrocytes has been reported to involve in the expression of inflammatory genes. It has been well established that the regulation of iNOS expression is mediated via tyrosine kinases such as JAK, MAPKs, ROS, and various transcription factors including STAT-1, NF-*κ*B, and AP-1 in astrocytes [131]. Increasing evidence suggests that activation of signal transduction pathways like c-Src, PI3K/Akt, and MAPK cascades contributes to activation of astrocytes and microglia, leading to expression of inflammatory proteins and advanced damage in neurodegenerative diseases [25, 26, 135].

### 5.5. Adhesion Molecules

Cell adhesion molecules play an important role in inflammatory responses. Leukocytes continuously circulate throughout the body in order to come in contact with antigens sequestered within tissues. To enter tissues, circulating leukocytes migrate from the blood between vascular endothelial cells and into the tissue [136]. During this migration, leukocytes initially bind to endothelial cells via low-affinity adhesion molecules. The low-affinity adhesion in combination with the force of the blood flow results in rolling leukocytes on endothelial cells. Subsequently, adhesion molecule affinity is upregulated and leukocytes firmly adhere to the endothelium [136]. Finally, bound leukocytes migrate between the endothelial cells and into the tissue. The vascular cell adhesion molecule 1 (VCAM-1) is one of the inducible cell transmembrane glycoproteins of the immunoglobulin supergene family expressed on several cell types and plays an important role in a number of inflammatory and immune responses [137]. It was first identified as an adhesion molecule induced on endothelial cells by proinflammatory cytokines or LPS [138]. VCAM-1 expression is induced on endothelial cells during inflammatory bowel disease, atherosclerosis, and infections [139]. Upregulation of VCAM-1 expression on cytokine-triggered vascular endothelial cells enhances the targeted transmigration of PMNs into extravascular space of inflammation [137]. In brain, proinflammatory cytokine-mediated expression of cell surface adhesion molecules plays a key role in endothelial cell injury, leading to vascular inflammation and the development of many cerebrovascular diseases [140]. Moreover, astrocytes can be induced by viral infections to express the adhesion molecules. Upregulation of adhesion molecules such as ICAM-1 (intercellular adhesion molecule 1) and VCAM-1 in astrocytes is required for monocyte-astrocyte interaction which increases infiltration of monocytes into the CNS observed in the patients with HIV-1 dementia [141]. HIV-1 Tat enhances monocyte adhesion by upregulation of ICAM-1 and VCAM-1 genes via a ROS-dependent NF-*κ*B activation in astrocytes [141]. Understanding the role of ROS in proinflammatory factor-mediated adhesion molecule expression and subsequently increased adhesion of monocyte to brain cells provides an occasion for the development of anti-inflammatory compounds that may be useful as therapeutic strategies for the CNS inflammation and ROS-associated neurotoxicity.

### 5.6. Stress Protective Proteins

In contrast with inflammatory proteins, recent reports indicate that the ROS can also induce several stress protective proteins, such as HO-1 and heat-shock proteins (HSP70 in particular), which may exert protective effects from the deleterious effects of inflammation [142]. Abnormal protein folding has been shown as a cause of various diseases like neurodegenerative diseases in association with inflammatory mechanisms. In the events, the HSPs play a crucial role in preventing protein misfolding and inhibiting apoptotic activity and represent a class of proteins potentially involved in PD pathogenesis [143]. Recent studies have shown that HSPs are colocalized in protein aggregates in AD, PD, and other neurodegenerative disorders [144, 145]. Many experimental findings have demonstrated that selective overexpression of HSP70 prevents the disease progression in various animal models and cellular models [145]. Furthermore, HSP70 dysfunction activates intracellular signaling like NF-*κ*B that can also promote neurodegeneration [146]. Thus, the expression of HSP70 may prove diagnostic and prognostic values in inflammatory conditions and therapeutical applications are being considered on the basis of these reports.

## 6. Redox Signal-Mediated Signaling Transduction

Recently, increasing evidence has demonstrated that oxidative stress (ROS generation) also plays a key signaling molecule in regulation of various inflammatory mediators in several cell types. Although many cells from brain tissue can produce various inflammatory mediators [42, 105], the intracellular signaling mechanisms responsible for the regulation of diverse inflammation-relating mediators expression induced by proinflammatory factors in brain cells like astrocytes are not completely characterized. Next, we review some signaling molecules in several inflammatory target protein expressions induced by proinflammatory factors in brain cells.

### 6.1. Mitogen-Activated Protein Kinases

Many proinflammatory cytokines and chemokines transducer signals are mediated via activation of MAPKs pathways. There is growing evidence that members of the MAPK family may play a central role in neurodegeneration [147]. MAPKs are important components of signaling modules activated by neurotransmitters, cytokines, and growth factors, as well as chemical and mechanical stressors. In mammals, three groups of MAPKs have been identified: the extracellular signal-regulated protein kinases (ERKs), the c-Jun NH_2_-terminal kinases (JNKs), and the p38 MAPK. ERK is activated by diverse stimuli, including growth factors and cytokines [147]. The p38 MAPK is activated by cellular stresses, including cytokines, LPS, growth factors, and UV radiation. The JNK is activated by many of the same stimuli that activate p38 MAPK, such as cellular stresses and various cytokines. Moreover, abnormal MAPK regulation might be implicated in CNS injury and inflammation [148]. Several mediators such as BK have been reported to act as an important proinflammatory factors through activation of MAPK cascades in different cell types [21–26]. In brain cells, the activation of ERK1/2 is mainly associated with proliferation, differentiation, and development in response to nerve growth factors. In contrast, the JNK and p38 MAPK signaling pathways are activated by various environmental stress and inflammatory factors that have been shown to promote neuronal cell death [149]. Moreover, the JNK and p38 MAPK signaling cascades can also be strongly activated by stress-induced ROS production or a mild oxidative shift of the redox state [28]. Both JNK and p38 MAPK are recognized as contributors to neurodegeneration by their ability to mediate intracellular stress events in transgenic mouse models of AD [19]. The p38 MAPK activation and COX-2 and PGE_2_ induction are served as contributors to neuronal damage in AD in response to oxidative stress [150].

In nonneural cells like astrocytes, many studies have found that A*β* peptide can activate astrocytes, including morphological alterations, cytokine induction, NO release [151], and chemokine and matrix-degrading proteinases production [152]. These findings further indicate that induction of several inflammatory mediators by the A*β*-stimulated activation of MAPKs in glial cells may be involved in AD progression. Moreover, our recent reports in astrocytes have demonstrated that the proinflammatory factors including TGF-*β* and BK can induce many inflammatory mediators such as MMP-9 expression through the ROS-dependent MAPK cascades [40]. These results suggest that upregulation of inflammatory mediators via ROS-mediated activation of MAPKs in astrocytes might play a key role during the CNS inflammation and neurodegeneration. Moreover, these results also implicate that the distinct groups of MAPKs are activated by a ROS-dependent manner which contribute to the expression of various inflammatory genes and are dependent on the external stimuli during brain inflammation. Thus, ROS may mediate MAPKs activation and expression of inflammatory genes in response to proinflammatory mediators in the CNS inflammatory disorders (Figure 5).

### 6.2. Transactivation of Receptor Tyrosine Kinases

Cross-communication between different signaling systems allows the integration of the great diversity of stimuli that a cell receives under varying physiological situations. The most direct mechanism is receptor heterodimerization that is well described for members of the epidermal growth factor receptor (EGFR) family [153]. In addition to growth factor receptor tyrosine kinases (RTKs) cross-talk, also completely unrelated cell surface receptors are able to communicate and influence each other, which play a key role in the transmission of information from outside the cell into the cytoplasm and nucleus. A variety of cytokines and growth factors that act as respective receptors have been reported to induce production of ROS in nonimmune cells. The prototype for such a pathway is the GPCR-induced transactivation of EGFR signal [154]. Treatment of cells with GPCR agonists induces phosphorylation of the EGFR by metalloprotease-dependent release of EGF-like ligands such as HB-EGF, thereby coupling GPCRs to EGFR characteristic downstream signaling pathways such as MAPKs or PI3K/Akt pathway [155]. In addition to the EGFR, other RTKs have been shown to be activated in response to GPCR stimulation, comprising the Trk receptor [156] and platelet-derived growth factor receptor (PDGFR) [157]. Previous studies have shown that in developing carcinoma cells, the early effects of COX-2-derived PGE_2_ and lysophosphatidic acid are in part mediated by the EGFR or PDGER, and this transactivation is responsible for subsequent downstream effects including the stimulation of cell migration and invasion [158]. However, receptor cross-talk can also occur in a ligand-independent manner involving for instance, non-RTKs such as c-Src [159]. Production of ROS results from the activation of signaling through the EGF and PDGF receptors [160]. In addition, ROS have been shown to stimulate c-Src-dependent transactivation of PDGFR*α* [161]. Accumulating evidence has shown that PKC-dependent activation of Nox is essential for PDGF-stimulated ROS generation, which is important for PDGF-induced MAPKs activation [162]. In the adult CNS, the EGFR pathway is highly upregulated and activated in astrocytes following neuronal injury [163]. Activation of the EGFR pathway triggers quiescent astrocytes to become reactive astrocytes that appear to be destructive to neurons in the adult CNS [163]. Regulation of RTKs such as EGFR in astrocytes may be a new therapeutic strategy for the treatment of neural disorders. These studies suggest that growth factor RTKs may play a pivotal role in mediating inflammatory genes regulation through ROS signal in several diseases including the CNS disorders (Figure 5).

### 6.3. Phosphoinositide-3′-Kinase (PI3K)/Akt Cascade

The phosphoinositide-3′-kinase (PI3K)/Akt cascade, the common downstream signal of EGF and PDGF receptors, is a cell survival pathway and regulated by various growth factor receptor-dependent mechanisms. Recent studies suggested that numerous components of the PI3K/Akt pathway play a crucial role in the expression and activation of inflammatory mediators, inflammatory cell recruitment, immune cell function, and tissue remodeling in chronic inflammatory diseases. In astrocytes, we demonstrated that ET-1 induced iNOS expression and NO production through PI3K/Akt cascade [26]. Moreover, PI3K/Akt cascade contributes to the expression of various inflammatory mediators induced by several proinflammatory factors in brain cells including astrocytes [125, 127]. Selective PI3K inhibitors such as wortmannin and LY294002 have been developed that reduce inflammation and some characteristics of disease in experimental animal models. In addition, ROS induction is often accompanied by the activation of PI3K/Akt cascade. For example, LY294002 has been shown to reduce chemokine-induced ROS generation in phagocytes [164], which was further confirmed by studies using PI3K knockout mice. Many studies have indicated the ROS generation induced by cytokines, PDGF, or VEGF in several cell types, which is reduced by inhibition of PI3K activity, suggesting that PI3K is involved in the ROS production induced by cytokines and growth factors. In addition to the role of PI3K/Akt cascade in ROS production, several reports support that the opposite hierarchical relationship exists between ROS and PI3K/Akt cascade. PI3K/Akt was activated in response to the exogenous treatment of H_2_O_2_ in several cell types [165]. Moreover, ROS have been shown to regulate phosphorylation of Akt [166] and then induce the expression of inflammatory genes associated with inflammation in various cell types. Taken together, these results implicate that ROS-dependent PI3K/Akt cascade or PI3K/Akt-mediated ROS signal may be critical for regulating the expression of inflammatory proteins in the brain inflammation and neurodegenerative disorders (Figure 5).

### 6.4. Transcription Factors

The progressive increase of oxidative stress during injuries not only causes oxidative damage to cellular macromolecules, but also modulates the pattern of gene expression through functional alterations of transcription factors. Here we focus on the roles of many transcription factors (e.g., NF-*κ*B and AP-1), which are well known to be modulated during oxidative stress associated with physiological and pathological events [32]. The transcription factors such as NF-*κ*B and AP-1 play a key role in the regulation of several gene expressions including proinflammatory cytokines, adhesion molecules, chemokines, growth factors, and inducible enzymes (e.g., MMPs, cPLA_2_, COX-2, and iNOS) during inflammation, immunity, cell proliferation, stress response, and apoptosis [167–169]. One important and widely investigated transcription factor which is NF-*κ*B is a major participant in signaling pathways governing cellular responses to environmental (oxidative) stresses [168]. The nuclear translocation and activation of NF-*κ*B in response to various stimuli, such as proinflammatory cytokines, LPS, and oxidative challenge (ROS production), are sequentially organized at the molecular level [168]. Moreover, NF-*κ*B act as a positive regulator in the expression of many inflammatory genes such as COX-2 involved in chronic inflammatory diseases [169]. Cytokines such as IL-1*β* and TNF-*α* have been shown to activate NF-*κ*B leading to upregulation of various NF-*κ*B-dependent genes in several cell types [168]. It is of interest that many of the genes regulated by these MAPK pathways are dependent on NF-*κ*B for transcription and lead to expression of inflammatory genes such as MMP-9 at the transcriptional level [169, 170]. In astrocytes, various stimuli can induce the expression of several inflammatory mediators, including MMP-9, cPLA_2_, COX-2, and iNOS, through ROS-mediated activation of NF-*κ*B manner [40, 62].

In addition, activator protein-1 (AP-1) is a sequence-specific transcriptional activator mainly composed of members of the Fos, Jun, and ATF-2 families. These proteins associate to form a variety of homodimers or heterodimers that bind to an AP-1 binding element within the promoter region of inflammatory genes such as COX-2 and MMP-9. It is a well-known redox-regulated transcription factor for the expression of several AP-1-dependent genes induced by diverse stress signals such as ROS generation associated with physiological and pathological events [25, 62, 170]. Several reports indicate that AP-1 is also involved in the pathogenesis of brain inflammation (Figure 5). Many studies have demonstrated that ROS signals (e.g., O_2_^∙−^ and H_2_O_2_) contribute to the expression or activation of AP-1 proteins (e.g., c-Fos) [62]. Recently, Kim et al. demonstrated that apocynin (a Nox inhibitor) shows potential antioxidant activities and inhibitory effects on the activation of redox-sensitive transcription factors, such as AP-1, induced by proinflammatory stimuli such as TNF-*α* [171]. The reports indicate that CSE induces cPLA_2_ expression through the production of ROS and subsequent activation of the MAPK pathway and AP-1 in human tracheal smooth muscle cells [172]. In astrocytes, we have demonstrated that AP-1 participates in the expression of several genes, including MMP-9 and HO-1, by BK through ROS-dependent manner [25, 62]. These results implicate that ROS play a central role in regulating AP-1 activation or expression and lead to inflammatory genes expression in brain inflammation and neurodegenerative disorders (Figure 5).

### 6.5. Transcription Coactivators

The transcription coactivator p300/CREB binding protein (CBP) is vital for the coactivation of several transcription factors such as NF-*κ*B and AP-1 in the transcription machinery, which has a significant role in the activation of transcription factor-mediated gene expression for proinflammatory factors [173–175]. The p300 protein is a key regulator of RNA polymerase II-mediated transcription. Several studies indicate that p300 participates in the expression of inflammatory genes induced by cytokines and growth factors. Furthermore, the transcriptional cofactor p300/CBP is an important component of the transcriptional machinery that participates in regulation at the levels of both chromatin modification and transcription initiation [173–175]. Previous studies have indicated that the promoter of several gene transcriptions, chromatin remodeling, and histone modification is regulated by p300/CBP [175]. However, in astrocytes, the p300 is vital for the coactivation of several transcription factors such as AP-1 in the transcription machinery, which has a significant role in the activation of AP-1-mediated gene expression for proinflammatory mediators [173]. Previous results have indicated that p300 plays an important role in BK-, IL-1*β*-, and oxLDL-induced MMP-9 expression in astrocytes [21, 22, 96]. Recently, a study has shown that ROS-dependent p300 activation leads to cPLA_2_ expression by cigarette smoke extract in human tracheal smooth muscle cells [172]. Consistently, we have demonstrated that LTA induces p300/AP-1-dependent MMP-9 expression via ROS-mediated pathway in astrocytes [27]. Moreover, oxidative stress activates NF-*κ*B resulting in the expression of proinflammatory mediators through the activation of intrinsic HAT activity on coactivator molecules. Oxidative stress also inhibits HDAC activity and in doing so enhances the expression of inflammatory genes which leads to a chronic inflammatory response. Oxidative stress can also increase complex formation between the coactivator p300 and the p65 subunit of NF-*κ*B suggesting a further role of oxidative stress in chromatin remodeling [1]. Together, these studies indicate that the oxidative stress-stimulated coactivator p300 may play a critical role in the expression of inflammatory genes during brain inflammation and neurodegenerative disorders.

## 7. Conclusions

**Figure 4:**
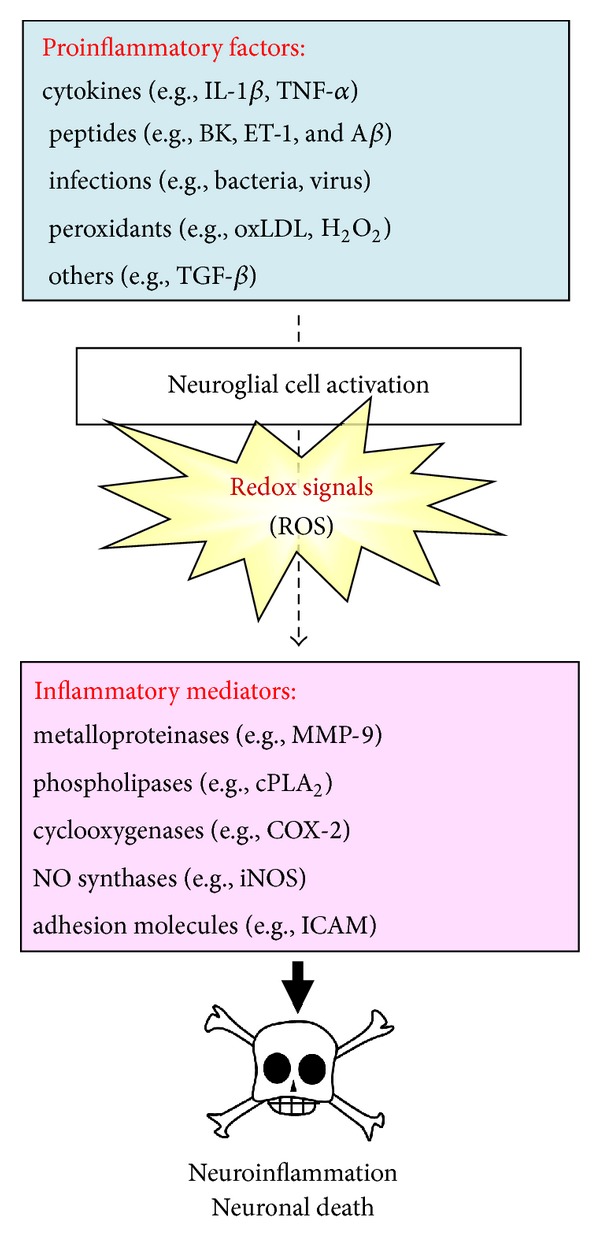
Schematic representation of the redox signals (ROS production) and their role in the development of neuroinflammation and neuronal death. Many of the well-known inflammatory target proteins, such as MMP-9, ICAM-1, VCAM-1, COX-2, and cPLA_2_, can be upregulated by various proinflammatory factors, including cytokines, peptides, bacterial or viral infection, peroxidants, via a ROS signal-dependent manner in neuroglial cells. These inflammatory mediators can cause neuroinflammation and neuronal death. IL-1*β*: interleukin-1*β*; TNF-*α*: tumor necrosis factor-*α*; BK: bradykinin; ET-1: endothelin-1; A*β*: *β*-amyloid; oxLDL: oxidized low-density lipoprotein; H_2_O_2_: hydrogen peroxide; TGF-*β*: transforming growth factor-*β*; MMP-9: matrix metalloproteinase-9; cPLA_2_: cytosolic phospholipase A_2_; COX-2: cyclooxygenase-2; iNOS: inducible nitric oxide synthase; ICAM: intercellular adhesion.

**Figure 5:**
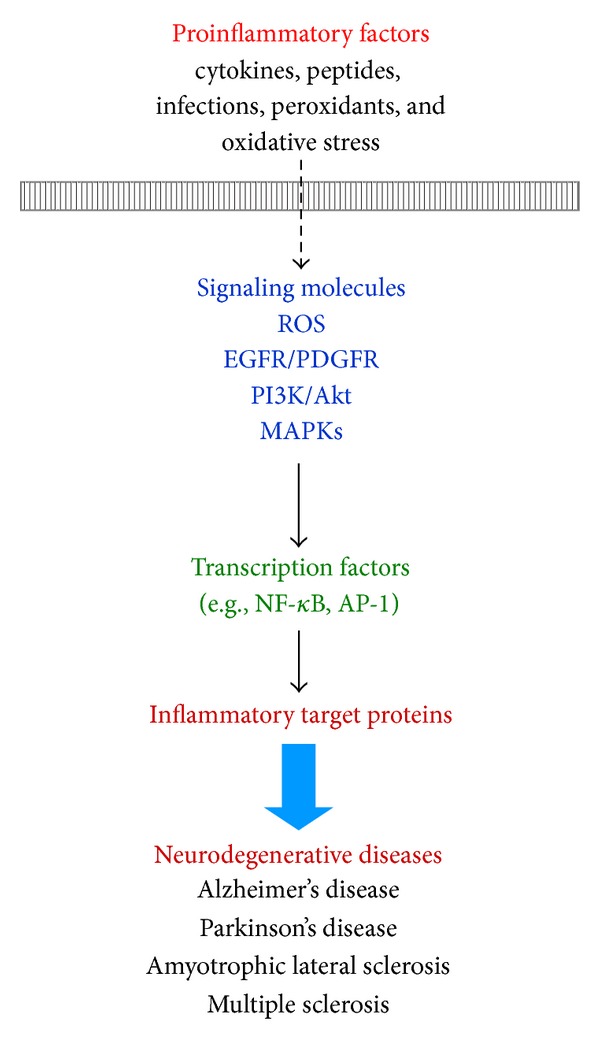
Proposed mechanisms of proinflammatory factors-stimulated activation of various signaling molecules and transcription factors leading to the expression of inflammatory target genes in brain resident cells. The intracellular signaling molecules include ROS, EGFR/PDFER, PI3K/Akt, and MAPKs. Oxidative stress may regulate these signaling pathways leading to activation of transcription factors such as NF-*κ*B and AP-1 and recruitment of coactivator p300 in the transcription initiation complex. Ultimately, upregulation of diverse inflammatory target proteins can cause the pathogenesis of several neurodegenerative diseases. EGFR: epidermal growth factor receptor; PDGFR: platelet-derived growth factor receptor; PI3K: phosphoinositide-3′-kinase; MAPKs: mitogen-activated protein kinases; NF-*κ*B: Nuclear factor-*κ*B; AP-1: activator protein-1.

Glial cells maintain brain plasticity and protect the brain for functional recovery from injuries. Reactivation of glial cells may promote neuroinflammation and neurodegeneration (Figure 1) and, ultimately, the retraction of neuronal synapses, which leads to cognitive deficits [10]. Moreover, redox signaling is a critical event in several inflammatory diseases such as AD that precedes the formation of these disease pathologies. To date, although numerous effects have been made to develop therapies based on antioxidants in the past years, the actual benefits to the patients have been very limited. It is likely due to lack of potency, late administration, and poor penetration into the brain cells [7, 32]. Alternative strategies including searching for factors that initiate endogenous antioxidants are necessary to improve the efficacy of treatment (Figure 2). Moreover, increased oxidative stresses (ROS) by various proinflammatory factors, such as cytokines, peptides, bacterial or viral infections, peroxidants, and other stress, serve as intracellular signals in gene regulation and signaling transduction, in addition to their deleterious effects on cellular components. Thus, understanding how oxidative stress produces and modulates expression of several genes that might help to develop effectively therapeutic strategies for CNS diseases. First, the focus of this review is on glial cells and their effects on the CNS disorders. Moreover, this review summarized the interplay between oxidative stress and neuroinflammation via ROS production which contributes to neurodegeneration, thereby enhancing disease progression based on data collected from brain cells, particularly astrocytes, in *in vitro* and *in vivo* studies (Figure 1). Perhaps modifying the activity of glial cells to reduce their neurotoxic properties and enhance their neuroprotective effects may offer potential targets for therapeutic interventions in neurodegenerative diseases. Oxidative stress-induced signaling transduction pathways, including ROS, transactivation of EGFR or PDGFR, PI3K/Akt, MAPKs, NF-*κ*B, and AP-1, that are associated with the CNS disorders were discussed (Figure 4). Moreover, the review highlighted current progress on the association of oxidative stress with the expression of various inflammatory genes, including MMP-9, cPLA_2_, COX-2, iNOS, and adhesion molecules and redox signal-sensitive transcription factors that may contribute to the development of the CNS inflammation and neurodegenerative diseases (Figure 5). Possible therapeutic strategies to target redox-sensitive signaling molecules, transcription factors, or cofactors are implicated based on the updated view of ROS-mediated regulation of inflammatory target genes in brain inflammation and neurodegenerative disorders.

## Abbreviations

ROS: Reactive oxygen species
CNS: Central nervous system
AD: Alzheimer's disease
PD: Parkinson's disease
MMPs: Matrix metalloproteinases
cPLA_2_: Cytosolic phospholipase A_2_
COX-2: Cyclooxygenase-2
Nox2: NADPH oxidase 2
iNOS: Inducible nitric oxide synthase
LPS: Lipopolysaccharide
IL-1*β*: Interleukin-1
TNF-*α*: Tumor necrosis factor-*α*
BBB: Blood-brain barrier
TLRs: Toll-like receptors
PGs: Prostaglandins
NO: Nitric oxide
A*β*: *β*-Amyloid
BK: Bradykinin
ET-1: Endothelin-1
oxLDL: Oxidized low-density lipoprotein
HO-1: Heme oxygenase-1
CO: Carbon monoxide
RNS: Reactive nitrogen species
Xox: Xanthine oxidase
GPCR: G-Protein-coupled receptor
LTA: Lipoteichoic acid
JEV: Japanese encephalitis virus
EV71: Enterovirus 71
AA: Arachidonic acid
VCAM-1: Vascular cell adhesion molecule 1
MAPKs: Mitogen-activated protein kinases
ERKs: Extracellular signal-regulated protein kinases
JNKs: c-Jun NH_2_-terminal kinases
EGFR: Epidermal growth factor receptor
RTKs: Receptor tyrosine kinases
PDGFR: Platelet-derived growth factor receptor
PI3K: Phosphoinositide-3′-kinase
NF-*κ*B: Nuclear factor-*κ*B
AP-1: Activator protein 1
CREB: Cyclic AMP-response element binding protein
CBP: CREB binding protein.
